# Assessing the knowledge of ethical clearance and animal welfare among researchers in Indonesia: A cross-sectional study

**DOI:** 10.14202/vetworld.2025.2499-2510

**Published:** 2025-08-30

**Authors:** Sutiastuti Wahyuwardani, Lisa Praharani, Susan Maphilindawati Noor, Bachtar Bakrie, Prima Mei Widiyanti, Wasito Wasito, Priyono Priyono, I. Gusti Ayu Putu Mahendri, Dimar Sari Wahyuni

**Affiliations:** 1 Research Centre for Veterinary Science, National Research and Innovation Agency, Jl. Raya Jakarta Bogor km 46, Cibinong, Jawa Barat, 16911, Indonesia; 2 Research Center for Animal Husbandry, National Research and Innovation Agency, Jl. Raya Jakarta Bogor km 46, Cibinong, Jawa Barat, 16911, Indonesia; 3 Research Center for Behavioral and Circular Economics, National Research and Innovation Agency, Jl. Gatot Subroto No 10, Kuningan, Jakarta Selatan, 12710, Indonesia; 4 Research Centre for Macroeconomics and Finance, National Research and Innovation Agency, Jl. Gatot Subroto No 10, Kuningan, Jakarta Selatan, 12710, Indonesia

**Keywords:** animal welfare, ethical clearance, Indonesia, Institutional Animal Care and Use Committee, research ethics, researcher knowledge, veterinary education

## Abstract

**Background and Aim:**

Ethical treatment of animals in scientific research is fundamental to ensuring data integrity and public trust. In Indonesia, the Institutional Animal Care and Use Committee (IACUC) plays a key role in ethical oversight, yet the extent of researchers’ knowledge regarding its roles and animal welfare (AW) principles remains unclear. This study assessed the level of understanding (UN) of ethical clearance and AW practices among researchers at the Indonesian Centre for Animal Research and Development (ICARD), focusing on variations based on educational background and professional position.

**Materials and Methods:**

A cross-sectional survey involving 107 researchers from: ICARD was conducted using a structured digital questionnaire assessing knowledge across three domains: IACUC roles, ethical clearance procedures, and AW implementation. Participants were stratified by educational background (veterinary vs. non-veterinary [NV]) and professional position. Non-parametric tests (Mann–Whitney U and Kruskal–Wallis) were used to evaluate group differences, with post hoc Dunn’s tests where applicable.

**Results:**

Veterinary researchers showed significantly greater UN of AW implementation (p < 0.01) and marginally higher knowledge of ethical clearance procedures (p < 0.10) compared to non-veterinarians. While IACUC knowledge was high across both groups, no significant differences were found (p = 0.161). By researcher position, prospective researchers demonstrated the lowest comprehension of AW practices (mean rank = 32.30), while junior researchers and research professors had the highest levels (mean ranks = 62.06 and 62.31, respectively). Position-based differences in IACUC and ethical clearance UN were not statistically significant, but significant variation was found in AW implementation (p = 0.035).

**Conclusion:**

This study reveals critical disparities in the UN of ethical clearance and AW among Indonesian researchers, particularly between veterinary and NV backgrounds and across researcher positions. Targeted ethics training, especially for early-career and NV researchers, is essential. Institutional policies should reinforce mandatory certification and continuous professional development to foster ethical research practices and enhance AW compliance.

## INTRODUCTION

Animal welfare (AW) is a fundamental ethical consideration in scientific research involving animals and has become a growing global concern. Researchers are increasingly expected to demonstrate both awareness and sensitivity regarding the humane treatment of animals used in research [1]. Research institutions bear a significant responsibility to ensure that AW principles are properly implemented at every stage of the research process. Adherence to these principles not only ensures ethical compliance but also enhances the quality and reliability of research outcomes [2].

Globally, the use of vertebrate animals in research and education is regulated by the Institutional Animal Care and Use Committee (IACUC), which is tasked with upholding ethical standards, ensuring legal compliance, reviewing animal use protocols, conducting inspections, and overseeing animal care practices [3]. In Indonesia, the IACUC was formally established by the Ministry of Agriculture in 2017 to standardize ethical oversight and promote responsible conduct in animal-based research.

Given the widespread use of animals in veterinary and animal husbandry research across Indonesia, assessing researchers’ knowledge and attitudes toward ethical clearance and AW is both timely and essential. While earlier studies have addressed AW principles and the critical role of IACUCs [4–7], most have focused primarily on ethical understanding (UN) within the social sciences. For instance, Drajati *et al*. [8] found that 59.4% of Indonesian researchers had limited knowledge of ethical considerations in social science research, underscoring the need for immediate academic reform. Similarly, Wardhono and Lestari [9] highlighted ongoing misunderstandings among researchers about the functions of Research Ethics Committees (RECs), despite general recognition of their importance. Internationally, Davis *et al*. [10] reported that many international students in Australia struggled with navigating ethical procedures, further emphasizing the need for training and support.

Some studies have reported improvements in awareness and UN of ethical processes. For example, Wardhono and Lestari [11] noted that social science lecturers were more knowledgeable about REC roles, while science and engineering faculty better understood procedural requirements. However, limited research has examined how well researchers understand ethical clearance in the context of animal-based studies – an important gap this study aims to address.

Despite the global emphasis on ethical standards in animal research and the establishment of IACUCs in many countries, including Indonesia, there remains a limited UN of how well researchers – particularly those engaged in animal-based studies – comprehend and implement these principles. Most existing research on ethics in Indonesian academia has centered on the social sciences, with studies highlighting general unawareness or misinterpretation of REC roles and ethical clearance procedures [8–11]. While these findings have prompted institutional reforms and academic interventions, they have largely excluded researchers working in veterinary, animal husbandry, and biological sciences – fields where animal use is most prevalent.

Furthermore, there is a paucity of empirical data evaluating how educational background (i.e., veterinary vs. non-veterinary [NV] training) and professional position (e.g., prospective, junior, and senior researcher) influence knowledge of AW principles and ethical review processes. Although IACUCs have been formally instituted in Indonesia since 2017, little is known about their effectiveness in disseminating ethical standards across different strata of the research community. Moreover, it remains unclear whether the growing emphasis on ethical oversight has translated into improved UN and implementation among early-career or NV researchers. This lack of focused investigation represents a critical gap, as inadequate knowledge in these areas can compromise research quality, violate regulatory requirements, and undermine AW.

This study aims to assess the level of UN of ethical clearance procedures and AW principles among researchers affiliated with the Indonesian Center for Animal Research and Development (ICARD). Specifically, it investigates whether researchers’ educational backgrounds (veterinary vs. NV) and professional positions influence their knowledge of the IACUC’s functions, ethical clearance protocols, and the practical implementation of AW standards. By identifying disparities in awareness and UN, this study seeks to inform targeted policy interventions, training programs, and institutional reforms. The ultimate goal is to strengthen ethical compliance and promote humane research practices across Indonesia’s animal research institutions, thereby enhancing both scientific integrity and AW.

## MATERIALS AND METHODS

### Ethical approval and informed consent

The ethical approval was not applicable to this this study; however, verbal informed consent was obtained from all participants. The study collected anonymous data through an online questionnaire, with voluntary participation and submission regarded as implied consent, in accordance with the Declaration of Helsinki 2013 and COPE guidelines.

### Study period and location

The study was conducted from January 2022 to April 2023 at the IACUC-ICARD in Bogor, Indonesia, involving participants from five institutions located in West Java, East Java, and North Sumatra.

### Sampling method

A purposive sampling strategy was adopted to recruit participants whose profiles aligned with the study’s objectives. Researchers affiliated with the ICARD, which comprises five research units across Indonesia, were invited to participate. Ethical considerations were strictly observed throughout the study, including informed voluntary consent, confidentiality, and avoidance of conflict of interest.

Three inclusion criteria guided the selection process: (1) Active employment at ICARD during the study period, (2) classification under one of two educational backgrounds – veterinary or NV, and (3) representation across a spectrum of professional positions, including prospective researchers (PR), assistant researchers (AR), junior researchers (JR), senior researchers (SR), and research professors (RP). This approach ensured diverse representation in terms of academic background and career stage.

### Sample size

**Figure 1:**
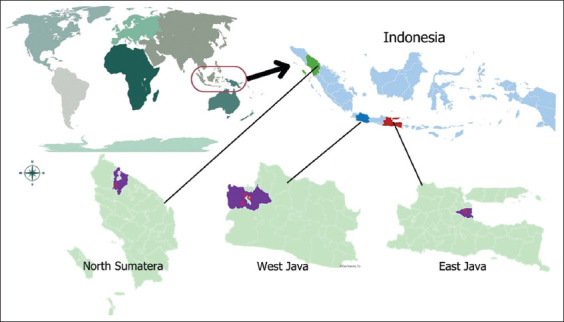
Map of the research area [Source: The map was generated using ArcGIS software].

From a total population of 138 researchers affiliated with ICARD, 107 were purposively selected to participate. This sample was considered sufficiently representative to meet the study’s analytical and inferential objectives [12]. Among the participants, 31 had veterinary training while 76 came from NV disciplines (Table 1). All five professional categories (PR, AR, JR, SR, and RP) were represented, with researchers distributed across ICARD research units in West Java, East Java, and North Sumatra (Figure 1). This geographic and institutional diversity provided insights into how researcher demographics may influence knowledge and practices regarding AW.

**Table 1:** Distribution of samples according to the researcher’s position level and educational background.

Position level	Sample size					

	Population (#researchers)			Sample (#researchers)		
	
	Veterinary	NV	Total	Veterinary	NV	Total
PR	4	7	11	2	8	10
AR	12	21	33	8	16	24
JR	14	22	36	10	16	26
SR	9	29	38	6	23	29
RP	7	13	20	5	13	18
Total	46	92	138	31	76	107

PR = Prospective researcher, AR = Assistant researcher, JR=Junior researcher, SR = Senior researcher, RP = Researcher professor, NV = Non-veterinary

### Data collection

A cross-sectional study design was employed. Data were gathered through a structured digital questionnaire administered through Google Forms. The questionnaire consisted of 28 statements designed to evaluate three core areas: (1) UN of the roles of the IACUC, (2) knowledge of ethical clearance procedures, and (3) implementation of AW principles in research.

Distribution was coordinated through ICARD unit heads, who shared the survey link with eligible researchers. This method ensured wide institutional coverage and encouraged participation from a broad range of professional and educational backgrounds.

### Observed parameters

The primary parameters observed in this study were respondents’ levels of knowledge and UN regarding:

- IACUC roles, including awareness of its objectives, governance, and regulatory functions in overseeing animal-based research;
- Ethical clearance, specifically UN of its purpose, the 3Rs principles (replacement, reduction, and refinement), and procedural compliance;
- AW implementation, measured by familiarity with the Five Freedoms (freedom from hunger and thirst, discomfort, pain or disease, fear and distress, and freedom to express normal behavior) as applied in research contexts [7].

### Statistical analysis

Descriptive statistics were used to summarize demographic characteristics and trends in UN. A standardized scoring rubric was applied to categorize knowledge levels into four bands: (1) Less UN (LU): X < 70, (2) UN: 70 < X < 80, (3) very UN (VU): 80 < X < 90, and (4) fully UN (FU): X ≥ 90 [13]. Data were analyzed using the Statistical Package for the Social Sciences version 26 (IBM Corp., Armonk, NY, USA) and R Studio (RStudio, PBC, Boston, MA, USA).

To assess differences in knowledge based on educational background (veterinary vs. NV), the Mann–Whitney U-test – a non-parametric method for comparing two independent groups – was applied [14]. The U statistics were computed using the following formulas:


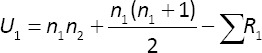



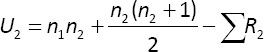


Where:

*n*_1_ = Number of observations in group 1

*n*_2_ = Number of observations in group 2

*R*_1_ = Ranks for observations in group 1

*R*_2_ = Ranks for observations in group 2.

The test hypotheses were as follows:

H_0_: No significant difference in UN between the groups.

H_1_: A significant difference exists between the groups.

To explore the association between researchers’ professional position and their level of UN, the Kruskal–Wallis H test was used, appropriate for assessing differences among more than two independent groups when assumptions of normality and homogeneity of variance are not met [15]. The test statistic was computed as follows:


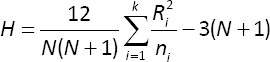


Where:

*N* = Total number of observations across all groups

*R_i_* = Sum of ranks for the i-th group

*n_i_* = Number of observations in the i-th group

The Kruskal–Wallis hypotheses were as follows:

H_0_: All groups have equal medians.

H_1_: At least one group differs significantly.

Where significant differences were detected, Dunn’s *post hoc* test with Bonferroni correction was used to identify specific between-group differences in AW implementation scores.

## RESULTS

### Effects of educational background

All veterinarians FU (100%) the roles of the IACUC (Table 2). Among NV respondents, 91.59% attained FU, with smaller proportions classified as LU (1.32%), UN (1.87%), and VU (5.61%). These results demonstrate generally high levels of IACUC knowledge among both groups, with veterinarians exhibiting marginally higher performance.

**Table 2:** Understanding levels of IACUC, ethical clearance, and AW implementation based on educational backgrounds.

Educational background	Parameter	Level of understanding (%)			

		LU	UN	VU	FU
Veterinary	IACUC	0.00	0.00	0.00	100.00
	Ethical clearance	41.94	0.00	58.06	0.00
	Implementation AW	3.23	12.90	54.84	29.03
NV	IACUC	1.32	1.87	5.61	91.59
	Ethical clearance	61.84	0.00	36.84	1.32
	Implementation AW	8.41	21.50	55.14	14.95

IACUC = Institutional Animal Care and Use Committee, AW = Animal welfare, LU = Less understanding, UN = Understanding, VU = Very understanding, FU = Fully understanding, NV = Non-veterinary

In terms of ethical clearance, the veterinarians displayed varied UN levels: 41.94% were categorized as LU and 58.06% as VU. No veterinarians were categorized as having UN or FU levels of UN. Among NV respondents, 61.84% fell into the LU category, 36.84% achieved VU, and only 1.32% attained FU, reflecting a lower overall UN of ethical clearance compared to veterinarians.

For AW implementation, veterinary respondents exhibited higher comprehension, with 54.84% and 29.03% of respondents classified as VU and FU, respectively. NV respondents were categorized as follows: 55.14% VU, 14.95% FU, 21.50% UN, and 8.41% LU. The majority showed at least a good UN of the AW. However, the results showed that veterinarians were more likely to achieve higher levels of comprehension.

No statistically significant difference was found between veterinary and NV respondents regarding the role of IACUC (Table 3). In the Mann–Whitney U-test, the mean ranks represent the average relative position of each group’s values in the combined, ordered data. A higher mean rank indicates that the group’s values are generally larger than those of the other group, suggesting a tendency toward higher outcomes. The Mann–Whitney U-test yielded a U value of 1,075.00, with p = 0.161, confirming that the two groups’ UN levels were statistically comparable.

**Table 3:** Mean rank of Mann–Whitney U-test statistics for the level of understanding based on educational background.

Variable	Educational background		U	Z	p-value

	Veterinary	NV			
IACUC	57.32	52.64	1,075.00	− 1.40	0.16^ns^
Ethical clearance	61.27	51.03	952.50	− 1.80	0.07*
Implementation AW	65.68	49.24	916.00	− 2.75	0.01***

IACUC = Institutional Animal Care and Use Committee, AW = Animal Welfare Committee, NV = Non-veterinary.

*** p < 0.01, **0.01 ≤ p < 0.05,

* 0.05 ≤ p < 0.10,

ns p ≥ 0.10

A marginally significant difference was observed in the UN of ethical clearance (U = 952.50, p = 0.073). Veterinary respondents (61.27) had a higher mean rank (p < 0.1) compared to non-veterinarians (51.03), suggesting a better comprehension among the former. However, the difference did not reach statistical significance at α = 0.05.

A statistically significant difference was identified in UN AW implementation (p = 0.006). Veterinary respondents achieved significantly higher mean ranks (65.68) than non-veterinarians (49.24), indicating a stronger UN. These findings reflect the superior comprehension of AW implementation among veterinary professionals.

**Figure 2:**
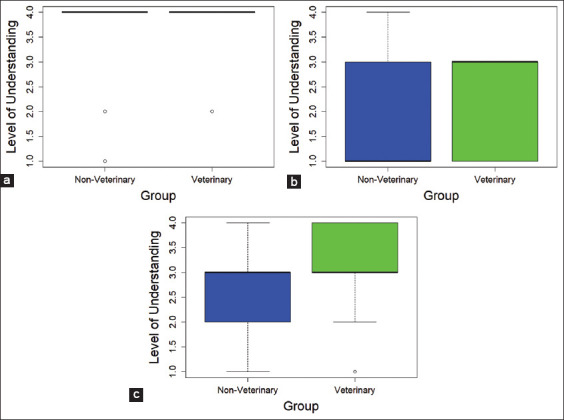
Boxplot comparing the level of understanding between the veterinary and non-veterinary groups using the Mann–Whitney U-test. (a) Institutional Animal Care and Use Committee. (b) Ethical clearance. (c) Implementation of Animal Welfare.

Boxplots further illustrate these findings (Figure 2). The statistical information in Figure 2, which includes medians, spread, and potential outliers, is already adequately presented in Table 3, containing the test statistics and p-values.

The boxplots for IACUC UN revealed comparable medians and interquartile ranges, corroborating the non-significant Mann–Whitney U-test result (p = 0.161). Figure 2a presents a boxplot comparing the level of UN of IACUC between respondents with and without veterinary education. Both groups exhibited the same median score of 4, indicating that most respondents in each group reported a high level of UN of IACUC. The NV group demonstrated greater variability, with two outliers scoring notably lower (1 and 2). In contrast, the veterinary group presented only one outlier with a score of 2. These findings suggest that the distribution of UN levels is more dispersed among NV respondents, although the central tendency is similar and the medians are identical.

For ethical clearance, the boxplots indicated slightly higher median and interquartile range values for the veterinary group compared with the NV group (Figure 2b). Despite the lack of statistical significance (p = 0.073), the visual trend suggests a modestly higher UN among veterinary respondents.

The boxplots in Figure 2c clearly distinguish between the two groups. The veterinary group displayed a higher median and lower variability than the NV group. This visual difference aligns with the statistically significant Mann–Whitney U-test result (p < 0.05), highlighting a better UN of veterinary researchers’ implementation of AW principles.

### Effects of the researcher’s position level

Table 4 presents only the proportions of UN levels by position of the researcher. The additional pairwise comparisons using Dunn’s test with Bonferroni adjustment, which is appropriate for non-parametric *post hoc* analysis following a significant Kruskal–Wallis test for differences among these groups, are shown in Table 5.

**Table 4:** Level of understanding of IACUC, ethical clearance, and implementation of AW based on various positions of researchers.

Position	IACUC (%)				Ethical clearance (%)				Implementation of AW (%)			
		
	LU	UN	VU	FU	LU	UN	VU	FU	LU	UN	VU	FU
PR	0.00	10.00	10.00	80.00	60.00	0.00	40.00	0.00	30.00	30.00	40.00	0.00
AR	0.00	4.17	8.33	87.50	50.00	0.00	50.00	0.00	8.33	25.00	50.00	16.67
JR	0.00	0.00	3.85	96.15	57.69	0.00	42.31	0.00	3.85	15.38	57.69	23.08
SR	0.00	0.00	3.45	96.55	58.62	0.00	37.93	3.45	3.45	27.59	65.52	3.45
RP	5.56	0.00	5.56	88.89	55.56	0.00	44.44	0.00	11.11	11.11	50.00	27.78
Total	0.93	1.87	5.61	91.59	56.07	0.00	42.99	0.93	8.41	21.50	55.14	14.95

IACUC = Institutional Animal Care and Use Committee, LU = Less understanding, UN = Understanding, VU = Very understanding, FU = Fully understanding, PR = Prospective researcher, AR = Assistant researcher, JR = Junior researcher, SR = Senior researcher, RP = Research professor, LU = Less understanding, AW = Animal welfare

**Table 5:** Kruskal–Wallis test results for the understanding level based on the position level of the researchers.

Variable	Position level	Mean rank	Kruskal-Wallis H	p-value
IACUC	PR	48.10	2.977	0.562^ns^
	AR	52.29		
	JR	57.00		
	SR	55.41		
	RP	52.94		
Ethical clearance	PR	51.70	0.441	0.979^ns^
	AR	57.00		
	JR	52.92		
	SR	53.24		
	RP	54.06		
Implementation AW	PR	32.30^a^	10.361	0.035*
	AR	53.08^ab^		
	JR	62.06^b^		
	SR	49.86^ab^		
	RP	62.31^b^		

IACUC = Institutional Animal Care and Use Committee, PR = Prospective researcher, AR = Assistant researcher, JR = Junior researcher, SR = Senior researcher, RP = Research professor. ***p < 0.01, **0.01 ≤ p < 0.05,

* 0.05 ≤ p < 0.10,

ns p ≥ 0.10.

^a, b, c^Different superscript letters within a column indicate statistically significant differences between treatment groups (p < 0.05)

The analysis of UN based on researchers’ positions indicated that most participants demonstrated FU, with JR and SR exhibiting the highest proportions at 96.15% and 96.55%, respectively (Table 4). RPs and AR also showed high levels of comprehension, with 88.89% and 87.50% achieving FU, respectively. PRs had comparatively lower FU rates, with 80% of the participants reaching this category.

For ethical clearance, the LU category was most prevalent among PRs (60%), followed by SRs (58.62%), JRs (57.69%), and RPs (55.56%). ARs exhibited the lowest proportion of LU at 50.00%, reflecting a relatively better UN of ethical clearance than other groups. VU was the predominant level among ARs (50%) and RPs (44.44%), whereas FU was rarely observed, except in 3.45% of SRs.

The majority of researchers demonstrated VU or FU level in the implementation of AW. SRs had the highest proportion of VU (65.52%), followed by JRs (57.69%), with ARs and RPs each at 50%. FU levels were highest among JRs (23.08%) and SRs (27.78%), whereas PRs had a notably lower representation in this category. LU was most frequently observed among PRs (30%), indicating the need to enhance their knowledge regarding AW implementation.

The Kruskal–Wallis test (Table 5) indicated no statistically significant differences in IACUC UN across researcher position levels (p > 0.05). The mean ranks were closely clustered, ranging from 48.10 for PRs to 57.00 for JRs. These findings suggest a comparable UN of IACUC principles across all positions.

Similarly, the Kruskal–Wallis test did not reveal a significant difference in UN ethical clearance across position levels (p > 0.05). The mean ranks were nearly equivalent, ranging from 51.70 (PR) to 57.00 (AR), indicating consistent levels of UN.

In contrast, a statistically significant difference was found in the UN of AW implementation (p < 0.05). RPs had the highest mean rank (62.31), closely followed by JRs (62.06), while PRs recorded the lowest mean rank (32.30). The results of the *post hoc* pairwise comparisons between different researcher position levels regarding the implementation of AW showed a statistically significant difference between PRs and JRs (p < 0.05). A significant difference was also found between PRs and RPs (Table 6). This suggests that JRs and RPs are more likely to differ in their implementation of AW than PRs. However, no significant differences were observed among ARs, SRs, and the other groups. These findings indicate that AW implementation comprehension varies significantly by position, with more experienced individuals, particularly RPs and JRs, demonstrating greater UN than PRs.

**Table 6:** *Post hoc* pairwise comparisons using Dunn’s test with Bonferroni adjustment of AW implementation.

Variable	Position level	Position level	Z-value	Adjusted *P* value (Bonferroni test)
Implementation AW	PR	AR	−1.968147	0.2453
	PR	JR	−2.850433	0.0218**
	PR	SR	−1.706941	0.4392
	PR	RP	−2.711659	0.0335**
	AR	JR	−1.130022	1.0000
	AR	SR	0.416072	1.0000
	AR	RP	−1.054214	1.0000
	JR	SR	1.609476	0.5376
	JR	RP	−0.028812	1.0000
	SR	RP	−1.478102	0.6969

PR = Prospective researcher, AR = Assistant researcher, JR = Junior researcher, SR = Senior researcher, RP = Research professor. ***p < 0.01,

** 0.01 ≤ p < 0.05, *0.05≤p < 0.10, ^ns^p ≥ 0.10

**Figure 3:**
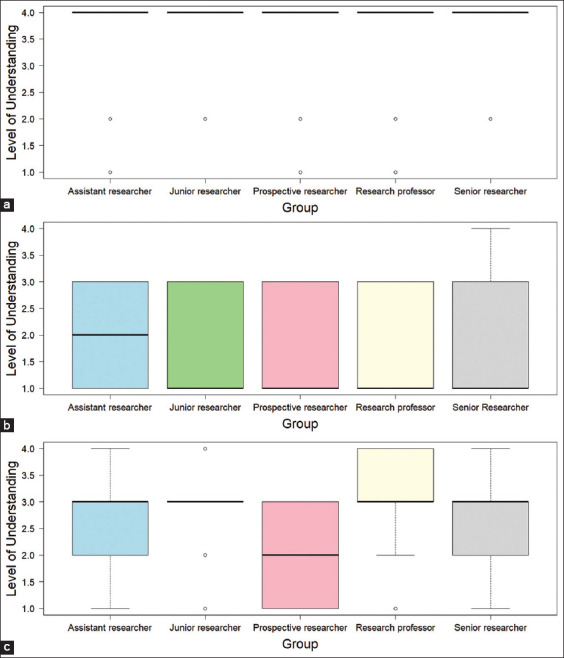
Boxplot comparing the level of understanding among researchers using the Mann–Whitney U-test. (a) Institutional Animal Care and Use Committee. (b) Ethical clearance. (c) Implementation of Animal Welfare.

Boxplots illustrate the distributions of UN across all positions, with similar medians and interquartile ranges for IACUC (Figure 3). This visual trend aligns with the Kruskal–Wallis test result, which found no statistically significant differences. Figure 3a displays a boxplot comparing the level of UN of IACUC among the five researcher positions. All groups reported a similar median score of 4, indicating that respondents at all researcher positions generally perceived a high level of UN. Despite the consistency in median scores, the boxplots reveal outliers in each group, with individual scores as low as 1 or 2. The visual evidence indicates minimal variation in central tendency across groups, with observable differences primarily related to the distribution spread and the presence of outliers.

Ethical clearance boxplots (Figure 3b) showed nearly uniform medians across PRs, ARs, JRs, SRs, and RPs, confirming the lack of significant positional variation. Conversely, AW implementation boxplots (Figure 3c) revealed substantial differences among position levels. PRs exhibited the lowest median and widest variability, whereas RPs and JRs exhibited higher and more consistent median values.

## DISCUSSION

### Overview and context

This study provides a nuanced UN of how researchers in Indonesia perceive and implement AW principles, highlighting key disparities based on educational background and professional level. Using data on knowledge of the roles of IACUC, ethical clearance, and AW implementation, the study demonstrated both strengths and gaps in the current research environment. The underlying frameworks – the Three Rs (replacement, reduction, and refinement) and the Five Freedoms – remain central to the ethical use of animals in research [16, 17] and must be effectively internalized by all who engage in animal-based studies.

This study evaluated the knowledge and awareness of AW among researchers in Indonesia. The assessment encompassed awareness of the IACUC, UN of AW principles, and their application in research settings. AW principles are grounded in the Three Rs (replacement, reduction, and refinement) [16] and the Five Freedoms (5F) framework, which includes freedom from hunger and thirst; discomfort; pain, injury, or disease; expression of normal behaviors; and fear and distress [17]. The principles of AW are grounded in the Three Rs (replacement, reduction, and refinement) [16] and the Five Freedoms framework. Public awareness of AW and its contribution to sustainability is vital for Indonesia’s long-term development goals [6, 18].

### Differences by educational background and research position

Veterinary researchers generally demonstrated a greater knowledge of AW, particularly in relation to IACUC functions. This may be attributed to curriculum structures that emphasize ethics and welfare in veterinary education [19]. In contrast, NV researchers showed weaker UN, especially regarding ethical clearance and AW implementation. This pattern mirrors prior findings in other fields, such as social science and international postgraduate research, where ethical awareness was also limited [8, 10]. The findings offer important insights into awareness of AW and its implementation in research across Indonesia, especially in the ICARD. Key areas for improvement are identified and the importance of training programs to strengthen researchers’ UN of AW is underscored.

The study revealed variations in comprehension levels, with veterinary researchers showing a higher degree of UN, especially concerning IACUC. However, NV researchers show lower awareness, especially in terms of ethical clearance and animal care and use. Differences were also observed across professional ranks, with junior and SRs exhibiting greater UN of AW implementation than PRs. Statistical analyses further highlight that educational background significantly affects knowledge of ethical clearance and animal care, while the researcher’s position also plays a crucial role in shaping this UN. These findings are consistent with those of Beaver and Golab [19], who noted that veterinary colleges are increasingly incorporating AW courses to enhance professional competencies. Blaxter [20] also noted that UN AW is based on values and science.

### Ethical oversight and global practices

Researchers bear a critical responsibility to treat research animals with respect, care, and consideration – an essential component of ethical and reliable scientific practices [21]. Ethics assessment requires ethical approval before allowing research involving animals [22]. Several countries, such as Australia, the United Kingdom, and various European nations, have established regulations to govern animal research [23]. These regulations emphasize ethical review and oversight to ensure that research animals are treated humanely. Adherence to these regulations is critical for maintaining research integrity and ethical standards. Compared to other countries, Indonesia is still refining its regulatory framework; although IACUCs represent progress, further measures are needed to ensure consistent implementation and compliance. IACUCs broaden ethical oversight and improve institutional capacity to address public concerns regarding laboratory AW [24].

### Current status and role of IACUC in Indonesia

The IACUC, established in Indonesian research institutions, represents a significant step in promoting ethical animal research [6]. Since 2017, the Indonesian Ministry of Agriculture has promoted IACUCs through annual outreach initiatives aimed at raising the awareness of researchers. This study revealed that most researchers, regardless of educational background or position, acknowledged the value of IACUCs in promoting ethical research. Researchers must collaborate with the IACUC to develop protocols that adhere to ethical standards and ensure compliance with approved procedures [25]. These findings suggest that continuing training and socialization efforts can significantly improve the knowledge of researchers regarding IACUC regulations [26]. Nevertheless, barriers, including limited resources, weak enforcement mechanisms, and insufficient institutional commitment, remain significant challenges.

### Ethical clearance: Awareness and challenges

Ethical clearance is fundamental to animal research, ensuring that ethical considerations are met before a study begins [27]. This study identified a notable gap in UN ethical clearance procedures among researchers at different career stages. Based on the annual report of IACUC in ICARD, with only 68% of research proposals obtaining ethical clearance, there is a clear need for enhanced awareness and educational initiatives. Drajati *et al*. [8] reported a similar lack of UN of ethical clearance and found that 59.4% of social science researchers did not include ethical considerations in their studies due to a lack of knowledge about ethical clearance requirements. This issue can be understood through workshops, seminars, and accessible resources. Institutions must adopt a proactive stance to ensure that ethical clearance becomes an integral component of research planning and not a procedural formality [28].

### Training and institutional strengthening

UN AW principles is essential [29]. This enables researchers to make informed decisions and adopt practices that promote AW in their studies. Integrating high AW standards into research improves data reliability and validity [30]. Ethical animal research training equips researchers with regulatory knowledge, promotes AW, and fosters responsible scientific conduct [31]. Therefore, ongoing training initiatives are vital for improving researchers’ UN of ethical considerations related to animal research. Furthermore, institutional collaboration with international bodies regulating ethical animal research can provide Indonesian researchers with valuable insights and best practices that can be adapted.

### Persistent gaps and cultural influences

The findings of this study suggest that researchers’ knowledge of AW remains similar regardless of their veterinary or NV background. This may be attributed to training and socialization efforts by the IACUC. However, some researchers, particularly those in certain functional positions, still lack a comprehensive UN of AW. This highlights the necessity of continuous education and training programs to reinforce the ethical responsibilities of researchers. Furthermore, factors such as the perceived necessity of the experiment, species used, gender, educational background, and cultural context influence public perception regarding the use of animals in research [32]. Adherence to humane treatment standards and ethical practices is vital for sustaining public trust in scientific research.

### Protocol implementation and systemic issues

The implementation of AW principles is reflected in the research proposals submitted for ethical approval. Researchers must detail their methodology, the number and species of animals, and procedures for animal care [33]. The IACUC reviews these protocols to ensure compliance with ethical standards, requesting modifications when necessary [34]. However, this study found that not all researchers fully understand the ethical clearance process, and some do not apply for ethical registration. Upholding ethical integrity in animal research necessitates a strong commitment from researchers to prioritize AW at all stages of the study [35]. Reinforcing this commitment calls for a coordinated effort among government agencies, research institutions, and funding bodies to establish a unified ethical animal research framework.

### Implications and recommendations

These findings point to several critical implications. First, the variation in UN among researchers calls for mandatory, structured ethics training tailored to different academic backgrounds and career stages. Such programs should emphasize real-world scenarios, regulatory expectations, and the scientific value of high welfare standards [29–31]. Second, institutions must redefine the role of IACUCs – not merely as oversight bodies, but as partners in education and practice. Studies such as those by Lee *et al*. [34] and Holthaus *et al*. [25] confirm that researcher engagement with proactive, well-resourced ethics committees leads to stronger compliance and better research outcomes.

### Ethical research as a professional imperative

This study highlights the importance of ethical considerations in animal research. Researchers must uphold professional ethics by conducting research with integrity, transparency, and accountability [36]. The data further reveal that the professional levels of researchers significantly influence UN. The JRs and SRs showed notably better comprehension of AW principles than PRs. This aligns with the literature suggesting that ethical competence is not only educational but also experiential [37, 38]. However, while many researchers appear familiar with the existence of the IACUC, practical adherence to ethical clearance remains inconsistent. As highlighted in institutional reporting, only 68% of research proposals based on the IACUC annual report in ICARD were granted ethical clearance, with no research activity cancelation for proposals with no ethical clearance, suggesting that ethical approval is treated more as an administrative formality than a core component of responsible research in some cases [9, 28].

### Future directions and policy needs

To improve awareness and adherence to ethical standards, training and education initiatives should be reinforced [37]. Institutions must also strengthen communication channels between researchers and regulatory authorities to clarify and enforce ethical requirements. Prioritizing AW enhances scientific integrity, fosters societal trust, and promotes ethical and humane research practices. Strengthening the UN and implementation of ethical principles (positive human-animal relationship) in animal research benefits the scientific community and society [38]. Future research should examine the long-term effects of ETPs and institutional policies on improving adherence to AW standards.

### Policy recommendations

Based on these specific findings, we recommend the following:

1. Mandatory AW certification for all NV researchers before animal study approval, focusing specifically on ethical clearance procedures where we found the largest knowledge gaps.
2. Structured mentorship programs requiring PRs to complete supervised animal care rotations with SRs.
3. Position-specific training curricula, with intensive ethical clearance training for NV researchers and advanced welfare implementation training for all position levels.
4. Annual competency assessments using validated tools, with remedial training for scores below established thresholds.

## CONCLUSION

This study provides critical insights into the knowledge and application of AW principles and ethical clearance procedures among researchers at the ICARD. The results demonstrated that veterinary researchers exhibited significantly higher UN in the implementation of AW (p < 0.01) and moderately higher knowledge in ethical clearance procedures (p < 0.10) compared to their NV counterparts. In addition, position-based differences were evident, with junior and SRs showing substantially greater comprehension of AW principles than PRs (p < 0.05). Notably, UN of the IACUC was consistently high across both educational backgrounds and professional levels, though variability in ethical clearance and AW implementation remained concerning.

The practical implications of these findings are twofold. First, the variation in knowledge highlights the need for structured, mandatory training programs tailored to both educational background and research rank. Second, institutions must integrate ethics education into early career development pathways and reinforce continuous learning across all levels to ensure ethical compliance and uphold research integrity. Strengthening researcher engagement with IACUCs, not just as a procedural requirement but as a collaborative partner in research, is essential for advancing humane animal research practices.

One of the strengths of this study is its comprehensive sampling strategy that captures a diverse cross-section of educational backgrounds and professional positions across five ICARD research units. This wide representation enhances the generalizability of the findings within similar institutional contexts. Furthermore, the use of validated tools and non-parametric statistical methods guarantees robust data interpretation.

However, this study is not without limitations. The data were collected exclusively from researchers within ICARD, potentially limiting broader applicability across Indonesia’s wider research ecosystem. In addition, the self-reported nature of the questionnaire may introduce response bias. The cross-sectional design also precludes causal inference.

Future research should expand the geographic and institutional scope by including other research centers, universities, and private institutions involved in animal-based studies. Longitudinal studies examining the impact of structured ethics training and institutional policy shifts on researcher behavior would further strengthen evidence-based interventions. Furthermore, qualitative assessments could uncover nuanced barriers to ethical adherence not captured in quantitative surveys.

While the establishment of IACUCs marks a positive step toward ethical animal research in Indonesia, significant knowledge gaps persist – particularly among NV and early-career researchers. Bridging these gaps requires sustained institutional commitment, policy innovation, and education. By prioritizing AW as an ethical and scientific imperative, Indonesian research institutions can align with international standards, improve research quality, and foster greater public trust in science.

## AUTHORS’ CONTRIBUTIONS

SW: Conceptualization, investigation, methodology, formal analysis, and writing – original draft. LP: Methodology, investigation, formal analysis, data curation, and writing – original draft. SMN: Conceptualization, methodology, investigation, collected secondary data, and writing – original draft. BB: Conceptualization, validation, collected secondary data, investigation, and writing – original draft. PMW: Software, validation, investigation, data curation, and writing review – editing. WW: Conceptualization, methodology, formal analysis, data curation, and writing – original draft. PP: Validation, formal analysis, collected secondary data, data curation, and writing review – editing. IGAPM: Software, validation, formal analysis, collected secondary data, and writing review – editing. DSW: Software, validation, collected secondary data, data curation, and writing review – editing. All authors have read and approved the final manuscript.
